# Aspartic acid racemization of root dentin used for dental age estimation in a Polish population sample

**DOI:** 10.1007/s12024-018-9984-8

**Published:** 2018-05-02

**Authors:** Katarzyna Wochna, Radosław Bonikowski, Janusz Śmigielski, Jarosław Berent

**Affiliations:** 1 Department of Forensic Medicine, Medical University of Lodz, Sedziowska 18a Street, 91-304 Lodz, Poland; 2 Institute of General Food Chemistry, Lodz University of Technology, Stefanowskiego 4/10 Street, 90-924 Lodz, Poland; 3 Social and Technical Department, State Higher Vocational School in Konin, Przyjazni 1 Street, 62-510 Konin, Poland

**Keywords:** Forensic odontology, Dental age estimation, Aspartic acid racemization, Root dentin, Gas chromatography-mass spectrometry, Methodology

## Abstract

Precise age determination of unidentified bodies and human remains is one of the essential tasks of forensic science. The aim of this study was to assess the applicability of using the enantiomeric composition of aspartic acid racemization in root and crown dentin for dental age estimation using a Polish population sample. Coronal and root dentin from four teeth groups from the mandible were studied using gas chromatography with mass spectrometry. The results demonstrated a very high correlation between the chronological age and enantiomeric composition in both of the dentin samples. Individual linear equations of root dentin with correlation coefficients between 0.96 and 0.98 and a standard estimation error of ±2.95–4.84 years validated the application of aspartic acid racemization as a significant practical contribution to everyday forensic medical practice. Discrepancies in methodological aspects and modifications that simplify the protocol are presented.

## Introduction

One of the key tasks of forensic odontology is the identification of bodies of unknown identity, which is necessary not only for procedural and legal reasons but also for humanitarian reasons. The central assumption of this process is to obtain a shortlist of people who are reported missing or as victims. In the case of unidentified bodies or remains, the first task is to establish the decedent’s group features, followed by the decedent’s individual features, such as sex and chronological age, with the latter being determined as precisely as possible. Chronological age is an extremely important identification parameter. Many methods for the estimation of chronological age on the basis of dentition are known [1]; however, there is no accurate and objective method currently in use in Poland. The results from dental studies based on different populations [2, 3] may need to be verified [4] and, if required, modified to meet Polish requirements [5, 6], as inter-population differences can arise.

The amino acid racemization reaction is a characteristic of each amino acid. Aspartic acid (Asp) racemization is a chemical method of dental age estimation that was introduced in the second half of the 20th Century and is currently one of the most accurate methods for the estimation of age [7]. Aspartic acid is an organic chemical compound and a protein amino acid that is involved in many human tissues, including tooth dentin. It is one of the amino acids that is most prone to racemization and has one asymmetric carbon atom, which makes it optically active. It has two enantiomers (optical isomers), which are mirror reflections of each other. Those enantiomers, L and D, can rotate in the plane of polarized light in opposite directions (left or right), which converts them into a racemic mixture [8, 9]. In other words, Asp racemization is a non-enzymatic reaction that causes age-dependent accumulation of a detectable protein product [10–12]. Living organisms predominantly contain the L form of the enantiomer, but over time, this form is partly converted into the D form. Death “stops” this conversion, as the racemization rate is temperature-dependent, both in vivo and postmortem. The Asp racemization rate measurement is the basic premise of accurate age assessment at the time of the individual’s death [13]. Gas chromatography allows for the determination of the quantitative ratio between the two isomers, which is subsequently used in calculations, though alternative laboratory techniques are also taken into consideration [14, 15]. Unlike many other conventional age estimation methods, this method is objective due to its chemical nature. It is not dependent on an examiner’s professional experience, but on a specific, quantifiable research procedure.

This investigation was undertaken to examine how precise, measurable and accurate this method is, whether the results obtained would be statistically significant, and whether this method could be practically integrated into the Polish forensic protocol for the identification of bodies or remains of unknown identity.

## Materials and methods

All teeth used in this study were extracted from 16 male cadavers who underwent medico-legal (judiciary) autopsies at the Department of Forensic Medicine at the Medical University of Lodz between the years 2012 and 2014. According to Polish law, this research can be conducted on the condition that formal consent from the Institutional Ethics Committee for Human Research (here, at the Medical University of Lodz) is obtained (Resolution No RNN/47/12/KE, 27.03.2012). Our department conducts autopsies for Lodz Province, which is in the center of the country. Autopsies on female cadavers are carried out approximately four times less often than male autopsies, which was the reason for using male teeth in this study; however, material from female cadavers was still collected. Bodies with a known metrical age of between 20 and 68 that were of Caucasian race with an unspecified Polish ancestry and full or partial dentition without any pathological changes (caries) or traces of dental treatment were included. We chose to sample the lower monoradicular teeth due to their regular anatomy and relatively easy preparation. These teeth include the central incisors (31, 41), lateral incisors (32, 42), canines (33, 43) and first premolars (34, 44). The tests were performed on root dentin and also on crown dentin and were analyzed separately (provided that both types were accessible). This paper presents the work conducted on root dentin. Maxillary teeth research is planned for future studies.

Examinations were conducted on 75 teeth, including 23 central incisors (21 samples of root dentin), 22 lateral incisors (14 samples), 16 canines (15 samples) and 14 first premolars (14 samples). In total, 64 samples were analyzed.

In our study, we used Ohtani and Yamamoto’s method [13] with the application of gas chromatography coupled with mass spectrometry (GC-MS); however, several of our own modifications were introduced (as presented in the Discussion).

The procedure consisted of three stages: the preparatory stage, laboratory stage and calculations.

### Preparatory stage

After extraction, the teeth were disinfected in a 10% formalin solution at room temperature for 24 h. The teeth were then gently dried, the enamel layer was removed from the tooth crown, and the cementum layer was removed from the root with a diamond bur and profuse water cooling. Longitudinal teeth sections were not made; instead, the teeth were dissected in the cementoenamel junction region using a diamond disc and substantial water cooling to obtain both root and crown dentin. The pieces of root dentin were dried and numbered. The samples were then washed sequentially with 0.2 M (approximately 0.7%) hydrochloric acid and double-distilled water three times in three separate vessels, followed by 96% ethyl alcohol and 99.5% diethyl ether for 5 min each. Next, the dried root dentin was pulverized in an agate mortar until dentin powder was obtained. The samples were then stored in 2 ml Eppendorf test tubes.

After assessing the initial results we determined that it was sufficient to prepare the samples only once rather than the three times that had been done in the early stages of the study. Initially, each pulverized dentin sample was divided into three equally weighted parts, which was not done in the latter part of the study.

### Laboratory stage

**Fig. 1:**
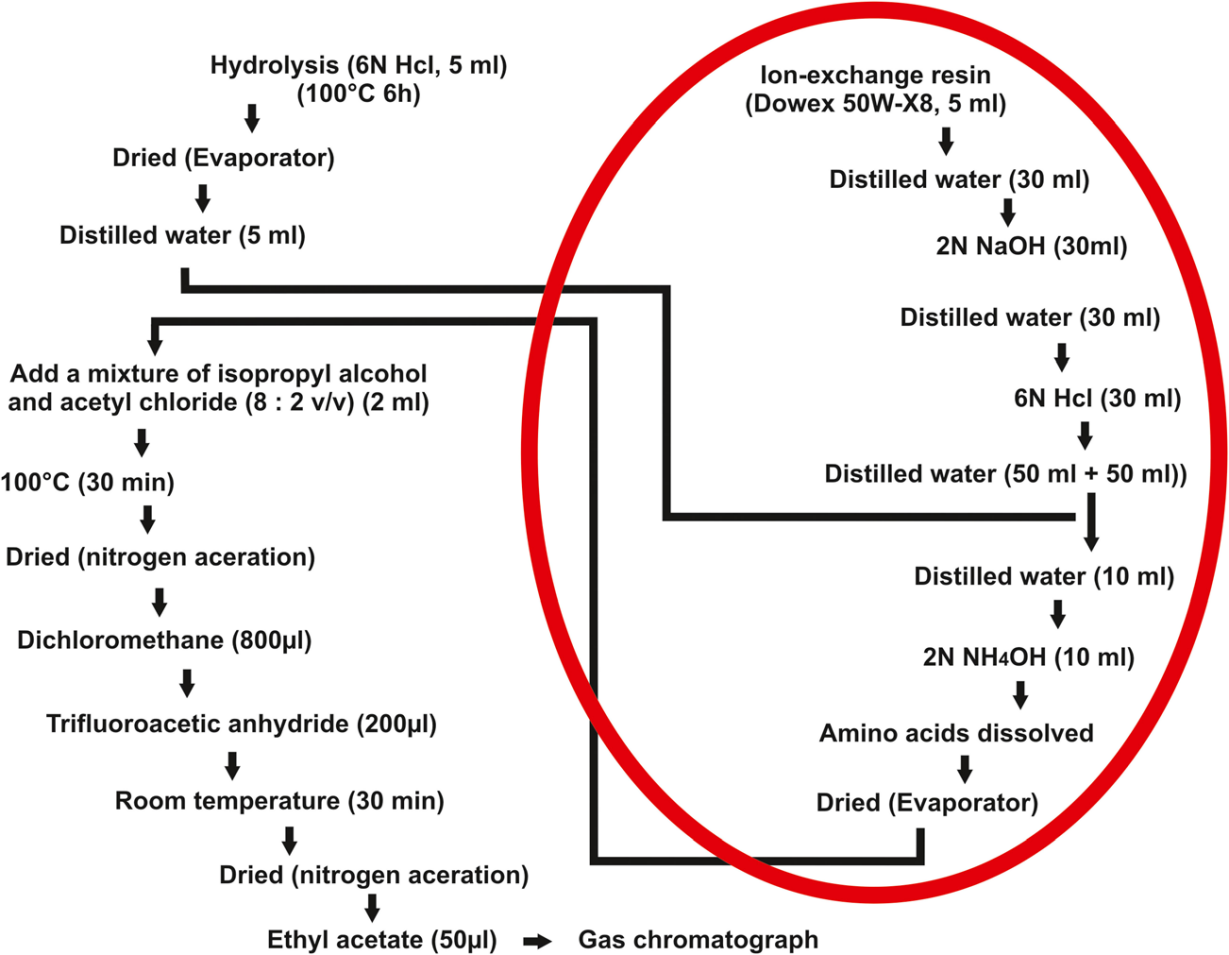
Scheme of the standard procedures developed by Ohtani and Yamamoto [13]. The red line indicates the omitted laboratory steps

The standard procedure for measuring the D/L ratio as developed by Ohtani and Yamamoto [13] is outlined in Fig. 1.

The procedure includes hydrolysis of pulverized dentin in 6 N hydrochloric acid, followed by sample drying, amino acid isolation via ion exchange chromatography, further sample drying, amino acid derivatization and gas chromatography (GC) analysis.

We eliminated the laborious stage of amino acid isolation and used a gas chromatography with mass spectrometry (GC-MS) method, which shortened the laboratory procedure (the stages marked in red on Fig. 1). This change to the protocol proved to be advantageous since it yielded results that were equivalent to those obtained in the original procedure, while also shortening the protocol length.

**Fig. 2:**
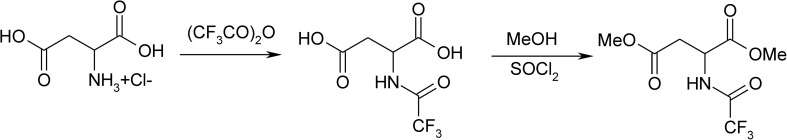
Scheme of the transformation of aspartic acid to N-trifluoroacetyl aspartic acid dimethyl ester

Thus, the following methodology was used. The sample was dried with a rotary evaporator at room temperature. Half a milliliter of trifluoroacetic anhydride was then added to the sample, which was then incubated for 1 h at room temperature. Next, the unreacted reagent was removed with a nitrogen stream, and 2 ml of a mixture of methanol and thionyl chloride (2:1 volume ratio) was added. The sample was then incubated for another hour at room temperature. Figure 2 illustrates this reaction. The sample was then transferred to a 50 ml flask to which 4 ml of ethyl acetate and 5 ml of saturated aqueous solution of sodium carbonate were added. After neutralization and separation of the layers, the organic portion was transferred to a vial, and 1 μl of the obtained mixture was analyzed by GC-MS. Each sample was analyzed in 10 repetitions, and the results that were outliers from the average (maximally four) were rejected.

Chromatography was carried out using an Rt®βDEXcst chiral column that was 30 m length, with a 0.32 mm inner diameter and 0.25 μm film thickness. Helium at a constant flow of 2 ml/min was used as the carrier gas. The injector temperature was 180 °C in split mode (1:100), the transfer line temperature was 220 °C, the ion source temperature was 200 °C, and the ionization energy was 70 eV. The mass spectrometer was operated in SIM (selected ion 158 and 198 monitoring) mode.

### Calculations

Initially, we used a linear regression model for the different types of teeth based on the work by Ogino et al. [16]. As the study progressed, however, a linear relationship between the metrical age and aspartic acid racemization rate was shown to exist, with correlation coefficients (R) ranging from 0.96 to 0.98. For this reason, we discontinued use the aforementioned model and instead used our own calculations. In addition, the previously used model was not reliable in our study because we used different study methods. Equations from linear regression analysis were newly developed for each examined tooth group.

### Statistical methods

Empirical distribution analysis with a normal distribution of the examined parameters was conducted using the Shapiro-Wilk test to verify whether further analysis would include parametric or non-parametric tests.

For each tooth group, i.e. the lower central incisors, lateral incisors, canines and first premolars, the regression equations, regression coefficients b_0_ (cross point of regression line with Y axis) and b_1_, standard error estimation (s), and correlation coefficients (R) were determined.

To characterize the average estimated age value, the arithmetical mean ($\overline{x}$) and median (Me) were calculated. The standard deviation (SD) was used as the measure of spread, and the ranges of the examined variables (the minimum and maximum values) were also specified.

To better present the empiric distribution, the asymmetry coefficient was also calculated.

The structure coefficients were calculated to assist with the description of the qualitative (unmeasurable) features of the examined groups.

Statistical analysis of the examined features (variables) was conducted using non-parametric tests, thereby analyzing the age distribution of the teeth groups.

Multidimensional statistical analysis for comparing the examined groups consisted of the non-parametric ANOVA Kruskal-Wallis rank test. This test was chosen because the distribution of the examined features was not consistent with a normal distribution. The Kruskal-Wallis rank test is a counterpart of the variance analysis test but meets the statistical assumptions for this type of variable analysis [17].

Box-and-whisker plots and boxplots were selected to present the results. Statistica 10.1 PL and Microsoft Office 2007 software were used for statistical and graphic data processing.

## Results

Table 1 shows the results of the multidimensional statistical analysis for each tooth group, which were not significantly different, indicating adequate material selection and that a further comparative analysis of the examined groups can be performed.

**Table 1:** Metrical age distribution in relation to the examined tooth group

Metrical age (in years)	Tooth group			
	Central incisors (Group 1)	Lateral incisors (Group 2)	Canines (Group 3)	First premolars (Group 4)
Number of samples	21	14	15	14
Minimal age	30.00	30.00	34.00	20.00
Maximal age	68.00	68.00	68.00	68.00
Median	50.00	48.00	50.00	43.50
Arithmetic mean	48.33	47.71	51.33	47.71
Standard deviation	14.72	14.68	13.33	16.29
Skewness coefficient	0.12	0.08	−0.03	0.02
Statistical analysis	Kruskal-Wallis test: H = 1.031 *p* = 0.793			

The results for each tooth group are presented in four tables (Tables 2, 3, 4, 5). In the first column, the sample number/tooth number (FDI numbering system) is presented; the second and third columns show the D form and L forms of aspartic acid, respectively; and the fourth column shows the natural logarithm of the [(1 + D/L)/(1 – D/L)] value. The last three columns show the metrical age, estimated age and ± error in years, respectively.

**Table 2:** Results for root dentin samples of central incisors

Sample number/ Tooth number	D	L	D/L	ln	Metrical age (in years)	Estimated age (in years)	± error (in years)
11/31	2,4	97,6	0,0246	0,0492	34	35	1
13/41	2,4	97,6	0,0246	0,0492	34	35	1
23/41	3,1	96,9	0,0320	0,0640	50	49	1
25/31	3,0	97,0	0,0309	0,0619	50	47	3
36/41	4,0	96,0	0,0418	0,0836	68	68	0
38/31	4,1	95,9	0,0428	0,0856	68	70	2
52/41	2,5	97,5	0,0256	0,0513	39	37	2
54/31	2,5	97,5	0,0256	0,0513	39	37	2
64/31	2,3	97,7	0,0231	0,0463	30	32	2
66/41	2,3	97,7	0,0236	0,0473	30	33	3
80/31	2,6	97,4	0,0264	0,0528	38	38	0
82/41	2,7	97,3	0,0275	0,0551	38	40	2
94/31	3,7	96,4	0,0379	0,0758	63	60	3
106/31	4,0	96,0	0,0413	0,0827	68	67	1
108/41	3,9	96,2	0,0400	0,0801	68	64	4
120/41	2,3	97,7	0,0234	0,0469	31	33	2
123/31	2,2	97,8	0,0224	0,0448	31	31	0
130/41	3,3	96,7	0,0337	0,0674	56	52	4
132/31	3,9	96,1	0,0404	0,0808	56	65	9
146/31	3,8	96,2	0,0394	0,0788	62	63	1
148/41	3,7	96,3	0,0382	0,0764	62	61	1

**Table 3:** Results for root dentin samples of lateral incisors

Sample number/ Tooth number	D	L	D/L	ln	Metrical age (in years)	Estimated age (in years)	± error (in years)
62/32	2,2	97,8	0,0222	0,0444	30	33	3
68/42	2,2	97,9	0,0220	0,0440	30	32	2
78/32	2,6	97,5	0,0262	0,0523	38	39	1
84/42	2,5	97,5	0,0261	0,0521	38	39	1
92/42	4,1	96,0	0,0422	0,0845	63	64	1
96/32	4,1	95,9	0,0426	0,0853	63	64	1
104/32	4,2	95,8	0,0440	0,0880	68	66	2
110/42	4,5	95,5	0,0473	0,0948	68	72	4
118/42	2,2	97,8	0,0221	0,0442	31	33	2
124/32	2,2	97,8	0,0225	0,0450	31	33	2
128/42	3,7	96,3	0,0387	0,0775	56	58	2
134/32	3,1	96,9	0,0316	0,0632	56	47	9
142/32	2,8	97,2	0,0290	0,0581	48	43	5
144/42	2,9	97,1	0,0302	0,0604	48	45	3

**Table 4:** Results for root dentin samples of canines

Sample number/ Tooth number	D	L	D/L	ln	Metrical age (in years)	Estimated age (in years)	± error (in years)
7/33	2,3	97,7	0,0240	0,0479	34	34	0
17/43	2,4	97,6	0,0244	0,0488	34	34	0
19/43	3,0	97,0	0,0306	0,0612	50	46	4
29/33	3,1	96,9	0,0316	0,0632	50	48	2
42/33	3,7	96,3	0,0386	0,0773	68	61	7
48/43	2,8	97,2	0,0285	0,0570	39	42	3
58/33	2,9	97,1	0,0297	0,0593	39	44	5
86/43	2,5	97,5	0,0257	0,0515	38	37	1
76/33	2,6	97,4	0,0267	0,0534	38	39	1
90/43	3,9	96,1	0,0408	0,0816	63	66	3
98/33	3,8	96,2	0,0397	0,0795	63	63	0
102/33	4,1	95,9	0,0430	0,0860	68	70	2
112/43	4,2	95,8	0,0436	0,0873	68	71	3
152/33	3,4	96,6	0,0355	0,0711	59	56	3
154/43	3,6	96,4	0,0371	0,0743	59	59	0

**Table 5:** Results for root dentin samples of first premolars

Sample number/ Tooth number	D	L	D/L	ln	Metrical age (in years)	Estimated age (in years)	± error (in years)
1/44	2,0	98,1	0,0199	0,0398	20	24	4
31/44	3,6	96,4	0,0378	0,0756	68	64	4
44/34	3,4	96,6	0,0353	0,0706	68	58	10
46/44	2,8	97,2	0,0286	0,0572	39	43	4
60/34	2,6	97,4	0,0271	0,0542	39	40	1
74/34	2,6	97,4	0,0268	0,0536	38	39	1
88/44	2,6	97,4	0,0266	0,0532	38	39	1
100/34	3,8	96,2	0,0396	0,0793	68	68	0
114/44	3,8	96,2	0,0397	0,0795	68	68	0
116/44	2,2	97,8	0,0226	0,0452	31	30	1
126/34	2,0	98,0	0,0203	0,0406	31	25	6
136/34	3,5	96,5	0,0365	0,0730	56	61	5
138/44	3,3	96,8	0,0336	0,0672	56	54	2
140/34	3,3	96,7	0,0343	0,0687	48	56	8

**Fig. 3:**
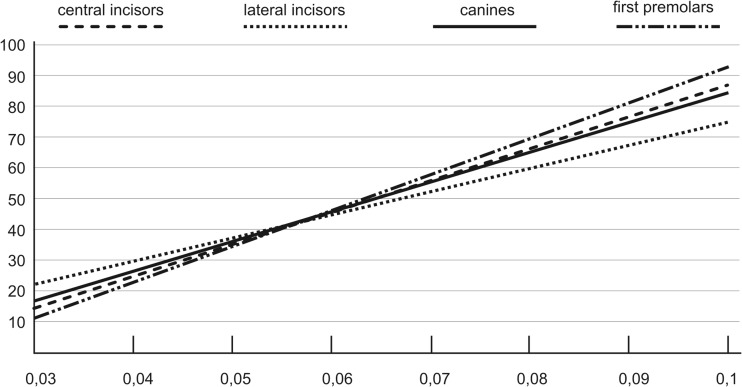
Illustration of four linear regression equations for metrical age (**Y**) dependence on the ln[(1 + D/L)/(1-D/L)] (**X**) parameter for root dentin in all the examined tooth groups

Table 6 presents our results in the form of linear regression equations for each tooth group for root dentin samples. Figure 3 is a graphic presentation of these equations.

**Table 6:** Linear regression equations for metrical age (**Y**) to ln[(1 + D/L)/(1–D/L)] (**X**) value relation (lower teeth, root dentin samples): n – number of analyzed cases; R–Pearson’s correlation coefficient; F –F statistics, verifying whole model significance hypothesis; s – standard estimation error (in years)

Metrical age relation to ln[(1 + D /L)/(1 – D /L)]	Linear regression analysis
central incisors	**Y** = 963.541 **X** – 12.747 *n* = 21; R = 0.98; F = 479.516; s = 2.947
lateral incisors	**Y** = 772.191 **X** – 1.574 n = 14; R = 0.97; F = 207.98; s = 3.570
canines	**Y** = 943.497 **X** – 11.529 *n* = 15; R = 0.97; F = 230.65; s = 3.195
first premolars	**Y** = 1123.305 **X** – 21.107 *n* = 14; R = 0.96; F = 135.44; s = 4.838

We found a very high correlation between the metrical age and enantiomeric composition of aspartic acid, i.e. the D/L form ratio. The strongest correlation was obtained for the central incisors, with *R* = 0.98, followed by the lateral incisors and canines, with *R* = 0.97, and first premolars with *R* = 0.96.

## Discussion

Several modifications were introduced in this study.

### Linear regression

As mentioned above, we established a new linear relationship between age and the racemization rate; we did not use the equations derived from the available literature [16], rather we created a novel methodology.

**Fig. 4:**
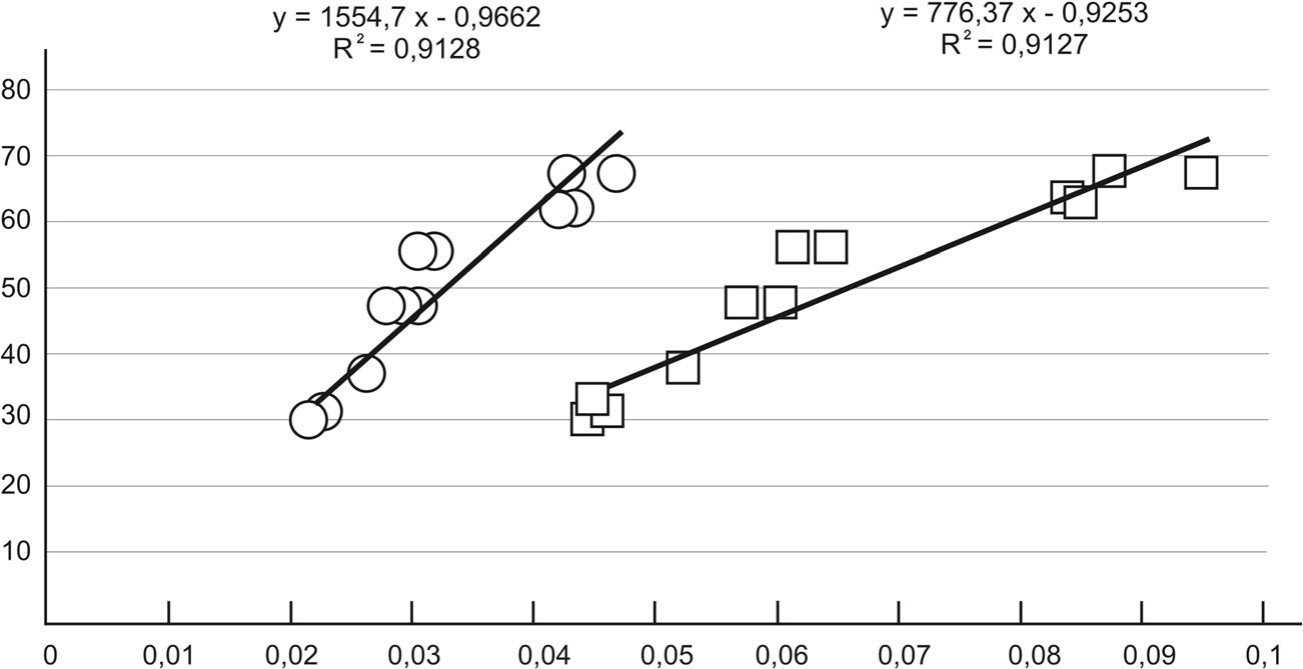
Illustration of the age = f (D/L) relation and age = f {ln[(1 + D/L)/(1-D/L)]} relation for lower lateral incisor root dentin samples. The Y axis is metrical age in years, and the X axis is D/L and ln[(1 + D/L)/(1-D/L)]. This figure is from the initial stages of the study

After testing each sample in SIM mode, we obtained the quantities of the D and L forms that were present in the samples. These values were used to calculate the D/L ratio for each sample. The D/L ratio was then applied to the following formula taken from previous studies: ln[(1 + D/L)/(1–D/L)] [13, 16]. We used this formula because when the age = f (D/L) relationship was applied, the resulting calibration line had a considerable slope, which resulted in an insignificant reading error of the surface areas under the chromatographic peaks of the analyzed substances which can generate a significant age reading error (Fig. 4). Thus we used the relationship of age = f {ln [(1 + D/L)/(1-D/L)]} instead, because in this case, the calibration line inclination angle is smaller, which compensates for the analytical error.

### Capillary column choice

The choice of an adequate aspartic acid derivative pattern confirmed that in research such as that presented here, a commercial capillary column with a chiral stationary stage (Rt®-βDEXcst) can be used successfully. Many companies provide commercial, i.e. mass-produced, columns. However, if needed, a column can also be designed and produced individually. In a previous study [13], the authors used an individually fabricated capillary column that was 15 m in length, with a 0.3 mm inner diameter. Stationary phase chirality was assured by L-*tert*-Leucine. Creating such a column is not only time-consuming but is also expensive, thereby increasing the material costs. Furthermore, the authors indicated that there were problems with creating a column that could provide excellent separation of enantiomeric forms. They also stated that they attempted to fabricate a capillary column that was able to completely separate D-Asp and L-Asp and clearly in a short period of time for accurate and simple testing. In a separate study, the same authors [18] noted that complete separation of the D- and L-forms in the chromatograms had been reported by only a handful of other researchers.

In our work, a complete separation of both enantiomers was also achieved and is presented below. An analysis of a racemate, which consists of an equal weight (50:50) of both enantiomeric forms, was conducted. If the chosen commercial column was not usable, we obtained values other than those mentioned, for example 45:55. In quantitative analysis of two aspartic acid derivatives using a stationary phase column, it is important to have a baseline separation between the analytes, as well as appropriately selected integration parameters that will allow the racemic mixture to achieve equal surface areas under the peaks of both analytes (due to possible differences in the peak shapes of the particular enantiomers) (Table 7).

**Table 7:** Calculations presenting a complete separation of both enantiomers, including the retention time in minutes, surface area in mV.s and surface area in %. Measurement parameters indicate that the desired equal surface areas under the chromatographic peaks of both analytes were obtained for the racemic mixture

	Retention time [min]	Surface area [mV.s]	Surface area [%]
1.	20.170	2.159	50.0
2.	20.533	2.155	50.0
	Total	4.313	100.0

To assess the usability of these modifications, an analysis of racemic aspartic acid that was converted into its N-trifluoroacetyl dimethyl derivative was performed. The Rt®βDEXcst chiral column was chosen based on its physical parameters and ease of accessibility. This column is 3 m in length and has a 0.32 inner diameter and stationary phase film thickness of 0.25 μm. Additionally, during the column testing stage, the following columns were analyzed: Cyclosil-β, Chiraldex-DA, Chiraldex-GA and Rt®-βDEXsm, although they did not produce acceptable results.

In summary, using a commercially available capillary column with a chiral stationary phase provides significant advantages as it reduces the workload and material costs and, most importantly, ensures the reproducibility of the method in cases in which column exchange becomes necessary.

### Sample weight

Our investigation showed that the weight of the sample plays an essential role in the quality of the results. We used samples of 10 mg or less (when the root dentin sample size after pulverization was less than 10 mg). This could occur, for instance, when the sampled tooth was small, for example a central incisor. These sample sizes were confirmed as appropriate by Ohtani and Yamamoto [13], who reported using 5-10 mg of dentin powder but also concluded that amounts as low as 2-3 mg were sufficient for analysis.

In the previously cited study, sample weights exceeding 10 mg of dentin of both types resulted in the analyte concentrations being too large and thus overloading of the chromatographic column. This led to imprecise results. The graphical representation of a particular chemical compound is shown by a chromatographic peak and is characterized by a considerably higher value than the chromatographic detector’s signal. When the column is overloaded, there is a significant peak tail, which enhances the surface area located behind it. This peak tailing distorts the peak shape, which should ideally resemble a Gaussian curve, which is a graphic description of a normal distribution.

Therefore, it was established that the peak shape is of crucial importance and could be influenced by two factors. The first factor, as mentioned previously, is a sample amount that is too large, and the second factor is wear-related damage of the column stationary phase. Prior to column replacement, the peak was stretched out, and after replacement, the peak was more consistent, resembling a Gaussian curve.

### Type of dentin and tooth group chosen for testing

In the majority of previous publications, the authors focused on examining crown dentin, but used different preparation techniques. For instance, Ohtani and Yamamoto [19] made longitudinal sections of the central incisors, whereas Ogino and Ogino [20], who also used crown dentin, analysed different teeth and did not section it. Ohtani [21] used root dentin from the central incisors, while Ritz et al. [22] utilized root dentin from the third molars.

Some authors examined both types of dentin, for example, Ohtani and Yamamoto [23] examined both types by using the second molars. These authors also published a study in which they prepared longitudinal sections of the central incisors and first premolars [24].

There is no apparent sampling strategy standardization [25]. Therefore, it was reasonable for us to use different teeth and both types of dentin in our research.

In previous publications, results have indicated a higher correlation with age in cases in which root dentin was used as the sample rather than crown dentin. Root dentin is considered to be less affected by diseases or repair processes (i.e. caries or reparative dentin deposition) [23, 25].

### Calculated age of the tooth

The tooth age, i.e. the end of its formation, is not identical to the chronological age of an individual because different teeth groups develop at different times. Studies of the time intervals necessary for complete crown and root tissue formation were carried out in the 1940s by Schour and Massler, who related chronological age to various stages of development and eruption of deciduous and permanent dentition; they published their outcomes as an atlas and charts [26, 27].

In one study [20], the crown completion ages of teeth were deduced from the Schour and Massler table and were used as correction factors. The age of an individual tooth was obtained by subtracting the aforementioned correction factor from the actual age of the person, and this was termed the “calculated age of tooth”, which refers to the age of the crown dentin.

It has been noted that few details of the Schour and Massler samples are known, but they were likely based on anatomical and radiographic sources [28]. Others have noted [29] that within the examined group of 29 people, 19 were below 2 years of age. Many other aspects of this atlas have been criticized, for instance, a lack of information about the analysis method that was used, the undefined developmental stages of teeth, and the small age group ranges [28–30]. It has also been discussed whether the given formation times represent the true age of the proteins that we would like to analyze, especially since the quantitative amino acid composition of these proteins is analyzed by racemization [22]. Additionally, in a review of the methodological aspects of aspartic acid racemization analysis [25], we found that some studies do not correct the dentin ages, which is acceptable if all of the examined teeth are of the same type, preferably from the same bone region, such as the mandible or maxilla [22]. This opinion is shared by Ogino et al. [16], who also observed that such teeth are likely to be chemically, structurally and developmentally more similar then teeth from different locations. In their opinion, it is not necessary to correct for crown completion age, so chronological age can be estimated directly from their regression lines, which are dedicated to different types of teeth.

The authors of the aforementioned review also specify that many problems can be avoided if individual calibration curves for each tooth type are constructed [25]. Both of these conditions were met in our study, and for these reasons, we did not use the calculated tooth age.

### Mass spectrometer (MS) scan mode

It is possible to use different working modes of the mass spectrometer depending on its type and capabilities. Two different modes include the SCAN and SIM modes [31, 32]. An analysis conducted in full scan (SCAN mode) presents the entire complexity of the multicomponent sample. By contrast, SIM (selected ion monitoring) mode is designed for selected scanning of particular ions. The advantage of SIM mode is that it eliminates unwanted analytes, which are not presented on the chromatogram, and it also increases the signal-to-noise ratio for the analyzed chemical compounds, resulting in improved precision and accuracy and positively impacting detection (sensitivity). For these reasons SIM mode was used in this study.

### Genetic and ethnic influence on aspartic acid racemization

Questions have arisen regarding the applicability of aspartic acid racemization to individuals who are genetically and/or ethnically dissimilar from the reference population. This issue must be examined in the context of the influence on variations in dentin’s protein composition. In one study [33], teeth from Turkish and German individuals with known ages were analyzed, and the relationship between aspartic acid racemization in dentin and age were identical in the two groups. The authors of this study concluded that considering these data and the literature, there is no indication of genetic and/or cultural influences on the protein composition of dentin or on the results of age estimation based on aspartic acid racemization in dentin. Another study [4] investigated ethnic differences in the racemization reaction velocities in which, after a heating experiment, the Arrhenius equation was used to obtain a rate constant (k). The results revealed that the racemization reaction rate velocities were nearly the same, indicating that there was practically no difference in the tooth substance of the two groups, as evidenced by the racemization reaction. Interestingly and related to issues mentioned above in the Discussion, due to the inclusion of several multiple-rooted maxillary and mandibular molars (14 of the 18 Scandinavian teeth, whereas 11 single-rooted Japanese teeth were used as the control), an accurate age estimation was not achieved in this study. After performing measurements of the racemization rates of aspartic acid using Japanese multiple-rooted teeth, it was confirmed that the type of teeth selected is extremely important for age evaluation by racemization. Other authors noted that when performing a pilot project evaluating the efficiency of HPLC coupled with fluorescence detection, the different racemization analytical results could be due to differences in the dentin samples as well as the analytical conditions of the various techniques [34], with no reference to the ethnic context. Another study investigated the application of aspartic acid racemization in age estimation in a Kuwaiti population using root dentin from 89 upper first premolar teeth. This work established a reasonably significant correlation between the D−/L-aspartic acid ratio and age and proposed an apparently reliable formula for calculating the age in Kuwaiti populations; however, they indicated that further research is required to ascertain whether similar findings are applicable to other ethnic populations [35]. To conclude, though the ethnic context may play role in the Asp racemization results, it appears that the most important factors may be those connected with sample selection and preparation as well as method standardization. This method has been analyzed from different perspectives with various methodological protocols, and further research could provide more information.

## Conclusions

The presented results demonstrate that application of an aspartic acid racemization method using root dentin allows for precise age estimates to be obtained. On the basis of linear regression models created in our initial studies, the estimated age was able to be calculated with high accuracy. Furthermore, the linear regression parameters confirmed that the credibility of such results was very strong. A nearly complete correlation between chronological age and enantiomeric composition was demonstrated. Application of this method, supported by the presented results, may be the foundation for using this method as an independent technique for dental age estimation, which is a significant practical contribution to the everyday forensic medical practice of estimating the age of bodies of unknown identity in Poland, particularly in Lodz Province. This initial study should, however, be repeated with different samples, not only from people from other Polish geographical areas but also with female and anterior maxillary teeth, to conclude whether this method is applicable to the entire Polish population.

## Key points

1. Aspartic acid racemization as a method of dental age estimation in a Polish population sample was assessed.
2. The applicability and accuracy of this method to the population sample that was studied was confirmed.
3. Application of this method using root dentin of lower anterior teeth allows for precise age estimates to be obtained on the basis of linear regression models created in initial studies.
4. The strongest correlation was obtained for the central incisors, with *R* = 0.98, followed by the lateral incisors and canines, with *R* = 0.97, and first premolars with *R* = 0.96.
